# Genetic Correction of the Most Common Mutation Causing Primary Hyperoxaluria Restores Enzyme Localization and Oxalate Metabolism

**DOI:** 10.1002/jimd.70122

**Published:** 2025-12-02

**Authors:** Timo Keskinen, Sami Jalil, Irem Gümüşoğlu, Juhana Juutila, Nadim Kestilä, Emilia Kuuluvainen, Ville Hietakangas, Diego Balboa, Kirmo Wartiovaara, Mervi E. Hyvönen

**Affiliations:** ^1^ Stem Cells and Metabolism Research Program, Faculty of Medicine, University of Helsinki Helsinki Finland; ^2^ Faculty of Biological and Environmental Sciences, University of Helsinki Helsinki Finland; ^3^ Institute of Biotechnology, Helsinki Institute of Life Science, University of Helsinki Helsinki Finland; ^4^ Clinical Genetics, Helsinki University Hospital Helsinki Finland; ^5^ New Children's Hospital, Pediatric Research Center, Helsinki University Hospital Helsinki Finland

**Keywords:** ABE, *AGXT*, alanine‐glyoxylate aminotransferase, base editing, CRISPR, inborn error of metabolism, inherited metabolic disease, lipid nanoparticle

## Abstract

Our research aimed to model primary hyperoxaluria type 1 in vitro using a stem cell model and assess the potential of adenine base editors in correcting the most common pathogenic *AGXT* genetic variant, c.508G>A (Gly170Arg), which leads to oxalate accumulation due to alanine‐glyoxylate aminotransferase mislocalization. Patient‐derived fibroblasts were induced to pluripotent stem cells, genetically corrected with adenine base editing, and subsequently differentiated into hepatocyte‐like cells in parallel with their non‐corrected isogenic counterparts. Enzyme localization was assessed through immunocytochemistry and confocal microscopy. The key metabolites associated with the disease were analyzed using liquid chromatography‐mass spectrometry to evaluate the metabolic phenotype. Finally, lipid nanoparticle formulations were designed and tested as an in vivo‐applicable delivery method for base editors. All induced pluripotent stem cell lines successfully differentiated into hepatocyte‐like cells and expressed essential hepatocyte markers, including *ALB, HNF1A*, and *AGXT*. Adenine base editor‐mediated genetic correction of the pathogenic *AGXT* mutation restored enzyme localization into peroxisomes and diminished oxalate accumulation without significant off‐target effects. Base editor mRNA and *AGXT* variant targeting single guide RNA encapsulated within lipid nanoparticles mediated gene correction in the hepatocyte‐like cell model. Using an in vitro model of primary hyperoxaluria type 1, we showed that base editor‐mediated genetic correction of the most common hyperoxaluria‐causing variant corrects enzyme mislocalization from mitochondria to peroxisomes and improves metabolic function. These results propose gene correction as a potential therapeutic approach to hyperoxaluria.

## 1 Introduction

**FIGURE 1:**
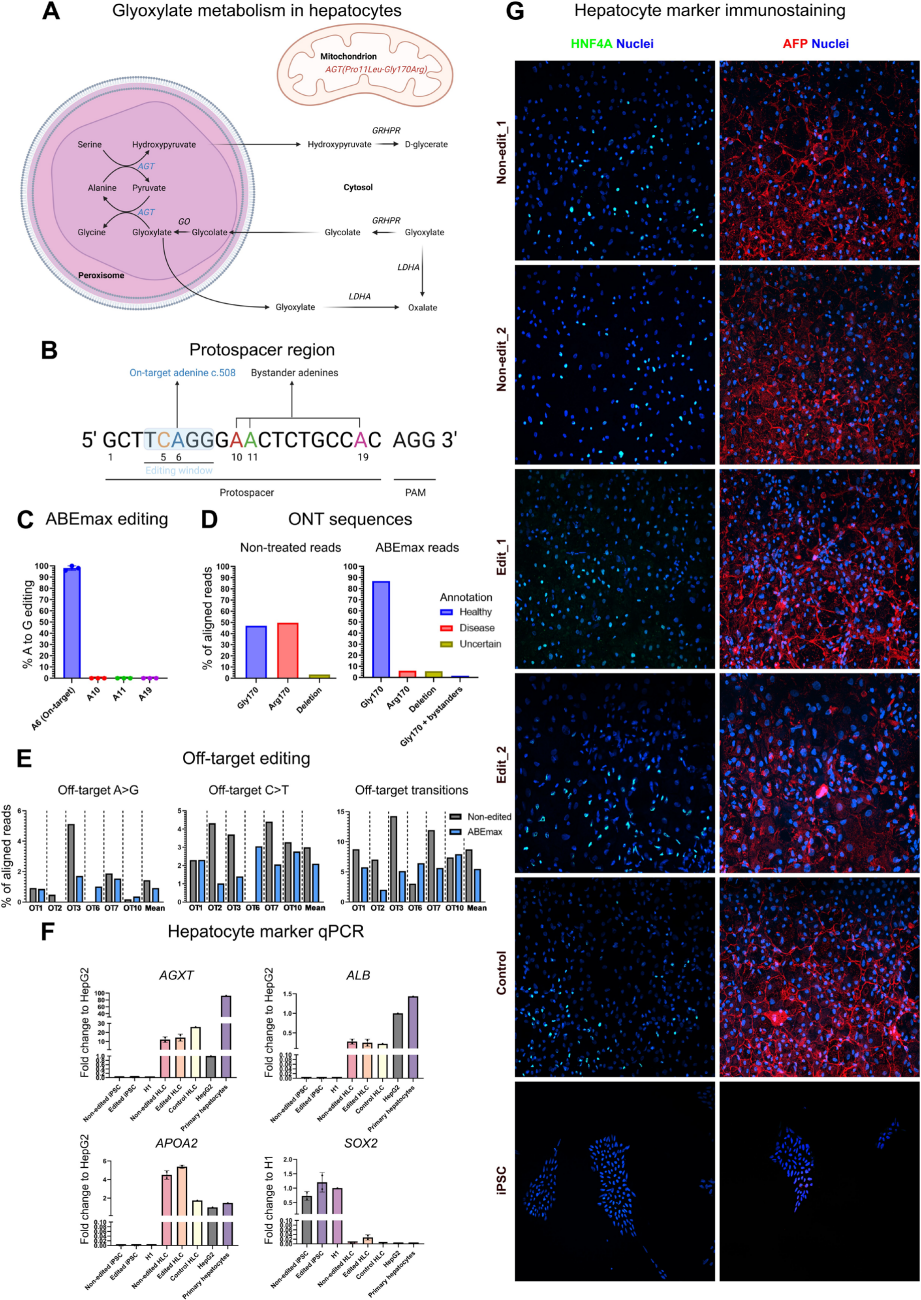
High‐efficiency base editing and iPSC line differentiation into hepatocyte‐like cells. (A) The pathway of glyoxylate metabolism in liver hepatocytes. The healthy AGT (*AGT*) localizes in the peroxisome and is depicted in blue. The AGT variant with both changes, Pro11Leu and Gly170Arg [*AGT(Pro11Leu‐Gly170Arg)*] depicted in red, localizes in the mitochondrion and is unable to participate in glyoxylate metabolism, which leads to PH1 and accumulation of glycolate, glyoxylate, and oxalate. GRHPR = glyoxylate reductase/hydroxypyruvate reductase, GO, glycolate oxidase (hydroxyacid oxidase); LDHA, lactate dehydrogenase. (B) The protospacer region for *AGXT_sg* and the on‐target editing of c.508. The protospacer region that the *AGXT*_sg (Table S1) targets is written from base 1 to base 20. The editing window (bases from 4 to 8) where the deaminase is the most active is highlighted in light blue. The on‐target adenine (base 6), which transition to guanine changes the Arg170 into Gly170, is depicted in blue. Bystander adenines in positions 10,11, and 19 are highlighted with red, green, and violet, respectively. The protospacer adjacent motif (PAM) needed for efficient guide binding corresponds to bases 21–23 (AGG). (C) On‐target ABEmax editing. PCR amplicons from three replicate (*n* = 3) fibroblast electroporation reactions were Sanger sequenced and analyzed for ABEmax editing of *AGXT* c.508 and bystander bases with EditR. Data are represented as the mean with SD. (D) ONT sequencing reads. The same electroporated DNA samples were pooled for ONT sequencing and analyzed with CRISPResso to get a deeper insight into the editing outcomes on an individual sequencing read level. The reads that aligned to the reference amplicon were analyzed based on the sequence in the 20 base pair long c.508 protospacer sequence and grouped into categories: “Gly170”, that includes all reads encoding the healthy Gly170 without additional base changes, “Arg170”, that includes all reads encoding the disease causing Arg170 without additional base changes, “Deletion”, that includes all reads with deletions, and “Arg170 + bystanders”, that includes all reads encoding the healthy Gly170 with additional base changes that do not lead to additional aminoacid changes. The categories were annotated “Healthy”, “Disease”, and “Uncertain” based on the predicted amino acid sequence of the reads of the category. The mean read count aligned to the reference amplicon was 371 for the on‐target samples. (E) Off‐target editing of ABEmax. The electroporated DNA samples were pooled for ONT sequencing and analyzed for off‐target editing with CRISPResso. All aligned off‐target reads were included in the analysis for increased sensitivity for rare off‐target events. The aligned reads were analyzed for the frequency of A>G substitutions, C>T substitutions, and total transitions (A>G, C>T, G>A, or T>C) in the top six off‐target protospacers OT1, OT2, OT3, OT6, OT7, and OT10 (Table 1) predicted by three independent software. The mean read count aligned to the reference amplicon was 400 for the off‐target samples. (F) mRNA levels of essential hepatocyte markers *ALB* and *APOA2*, pluripotency marker *SOX2*, and transcript of interest *AGXT*, in iPSC lines: Non‐edited (*n* = 2 independent cell lines), Edited (*n* = 2 independent cell lines), commercial embryonic stem cell line H1 (*n* = 1), differentiated hepatocyte‐like cell (HLC) lines: Non‐edited (*n* = 2 independent cell lines), Edited (*n* = 2 independent cell lines), and Control Hel24.3 cell line carrying two copies of the wild‐type *AGXT* allele (*n* = 1), HepG2 commercial hepatocarcinoma cell line (*n* = 1), and primary hepatocyte line (*n* = 1), analyzed by qPCR. The data are represented as a fold change to the HepG2 line, except for pluripotency marker *SOX2*, which is represented as a fold change to the H1 line. Data are represented as the mean with SD. (G) Representative immunocytochemistry images of day 20 hepatocyte‐like cells. Nuclear stain Hoechst is depicted in blue, hepatocyte marker AFP in red, and hepatocyte marker HNF4A in green. All images were acquired and processed with the same settings. This figure was created with BioRender.com.

Primary hyperoxalurias are autosomal recessive inborn errors of metabolism that lead to the accumulation of oxalate in various tissues. Primary hyperoxaluria type 1 (PH1, [MIM: 259900]) is the most common of primary hyperoxalurias with an incidence rate of 1 in 120000 live births in Europe [1]. PH1 results from pathogenic mutations in *AGXT* (MIM: 604285), which encodes liver‐specific peroxisomal alanine‐glyoxylate aminotransferase (AGT, [EC 2.6.1.44]). The AGT monomer forms a pyridoxal‐phosphate‐dependent homodimer, which catalyzes the transamination of glyoxylate to glycine in the liver hepatocytes [2] (Figure 1A). In PH1, the lack of catalytically active AGT in liver peroxisomes leads to the accumulation of glyoxylate that is converted into oxalate by lactate dehydrogenase (EC 1.1.1.27) [3]. The accumulating oxalate reacts with calcium and forms insoluble calcium oxalate crystals in the renal tubules. As the renal damage decreases the filtration rate, the kidneys cannot excrete the accumulating oxalate, which eventually saturates in the plasma and leads to systemic oxalosis [1, 4]. Until recently, PH1 treatment has comprised intensive hyperhydration, crystallization inhibitors, and pyridoxine supplementation. After kidney failure, a combined or sequential liver and kidney transplantation is the preferred treatment [5]. Novel therapeutical approaches disrupt the oxalate metabolism by gene‐targeted knockdown [6].

The most common pathogenic *AGXT* variant (c.508G>A [GenBank: NM_000030.3]; [Gly170Arg]; rs121908529) is found in 24%–37% of PH1 patients, is associated with significant catalytic activity, and leads to peroxisome to mitochondrion mistargeting [7, 8]. The variant is pathogenic when it is associated with the minor allele (AGT‐Mi) haplotype (c.32C>T [GenBank: NM_000030.3]; [p.Pro11Leu]; rs34116584). As a monomer, the AGT‐Mi N‐terminus adopts a conformation of an amphiphilic α‐helix able to act as a mitochondrial targeting sequence [9, 10, 11]. The healthy AGT dimerizes rapidly and irreversibly after synthesis, making it incompatible with mitochondrial import [12]. The Gly170Arg variant potentially acts synergistically with AGT‐Mi slowing down dimerization, thus keeping the mitochondrial targeting sequence unmasked [7, 13]. However, either Gly170Arg or AGT‐Mi variants alone do not promote significant enzyme mistargeting [12, 14].

CRISPR‐Cas9 base editors are precise genome editing tools that can be directed to correct transition changes in a narrow genomic locus. The adenine and cytosine base editors have collectively the potential to reverse most pathogenic point mutations [15]. Adenine base editors [16] (ABE) catalyze a base pair transition from A‐T to G‐C in the activity window and cleave the non‐edited DNA strand without leading to double‐stranded breaks, thus reducing the risk of genomic rearrangement, insertions, and deletions [17]. The base editors are being tested for their therapeutic potential in several clinical trials (ClinicalTrials.gov: NCT05398029, NCT05885464, and NCT05456880). Lipid nanoparticles (LNPs) facilitate mRNA transport into target tissues with low immunogenic effects compared to alternative delivery methods, such as viral vectors [18]. LNPs can be directed to different tissues by adjusting the LNP composition [19], but by default, they accumulate in the liver [20].

We hypothesized that *AGXT* mutations could be corrected by gene editing and proceeded to model PH1 with a genetic background of the Gly170Arg‐ and AGT‐Mi variants in vitro. Additionally, we tested the potential of base editors to correct the pathogenic mutation in differentiated hepatocyte‐like cells by an in vivo‐compatible LNP delivery.

## 2 Materials and Methods

### 2.1 Subject

An individual diagnosed with PH1 donated a skin biopsy sample for the research. The compound heterozygous patient carried *AGXT* variants c.508G>A (Gly170Arg) and c.32C>T (Pro11Leu) in one allele and c.673_676delAAGG in the other. The patient had a severe phenotype and presented with renal failure at the age of five months. The treatment comprised haemodialysis, peritoneal dialysis, pyridoxine, and sequential liver and kidney transplantations.

### 2.2 ABEmax And ABE8e In Vitro Transcription, Fibroblast Electroporation, and iPSC Generation

ABEmax and ABE8e in vitro transcription, fibroblast electroporation, and iPSC line generation were performed as previously described [21, 22].

In vitro transcription of ABEmax and ABE8e: The purified ABEmax [21] or ABE8e [22] IVT plasmid (Addgene: 201676 and 171761) was linearized by SfiI restriction (Thermo Fisher Scientific; FD1824). T3 RNA transcription was performed using the mMESSAGE mMACHINE T3 Transcription Kit (Invitrogen; AM1348) according to the manufacturer's protocol.

Fibroblast electroporation: One million fibroblasts were electroporated with the Neon Transfection System 100 μL Kit (Thermo Fisher Scientific; MPK10096). The electroporation parameters were as follows: three pulses, pulse width of 10 ms, 1650 V. The electroporated suspension contained 23 μg of in vitro transcribed base editor mRNA, 10 μg of single guide RNA, and 4 μM Alt‐R Cas9 electroporation enhancer (Integrated DNA Technologies; 1075916). For simultaneous pluripotent reprogramming, 1.5 μg of each of the three reprogramming plasmids, pCXLE‐hSK, pCXLE‐hUL, and pCXLE‐hOCT3/4 [23] (Addgene: 27078, 27080, and 27077) were added to the electroporation suspension.

iPSC line generation: Five days after the primary fibroblasts were electroporated with the reprogramming factors, they were seeded into Matrigel‐coated (Corning; 356231) six‐well plates at different cell densities. From this point, the medium was changed to hES [DMEM/F12 (Life Technologies; 31331‐028); supplemented with 20% KnockOut Serum Replacement (Life Technologies; 10828‐028); 0.0915 mM 2‐mercaptoethanol (Life Technologies; 31350‐010); 1% non‐essential amino acids (Life Technologies; 11140‐035); and 6 ng/mL bFGF (Sigma‐Aldrich; F0291)] supplemented with 0.25 mM sodium butyrate (Sigma‐Aldrich). Twelve days after electroporation, the medium was changed to Essential 8 medium (E8, Thermo Fisher Scientific; A1517001). From day 11 to day 17 after electroporation, the formed iPSC monoclonal colonies were manually picked from the fibroblast monolayer with a pipette and transferred to Matrigel‐coated 24‐well plates with E8 containing 10 μM rho‐associated protein kinase (ROCK) inhibitor (Y‐27632; Selleckchem). The iPSC lines were cultured on Matrigel‐coated 6‐well plates with E8 and passaged with 0.5 mM EDTA in PBS.

### 2.3 sgRNA Design and Manufacturing

The single guide RNA *AGXT*_sg was designed using the web tool Benchling (https://benchling.com). Integrated DNA Technologies manufactured the RNA (single guide RNA sequence in Table S1).

### 2.4 Determination of On‐Target Editing From Sanger Sequencing

Genomic DNA was isolated from a bulk population of cells and used as a template to amplify the genomic locus around the *AGXT* variant in a PCR reaction (PCR primers in Table S1). The resulting PCR product was purified, Sanger‐sequenced, and analyzed for on‐target editing using the web tool EditR [24].

### 2.5 Off‐Target Analysis From Sanger Sequencing

Three web tools, Benchling (Benchling [Biology Software] (2024). Retrieved from https://benchling.com.), Integrated DNA Technologies, and CRISPOR (http://crispor.tefor.net.), were used to predict the most likely off‐target loci across the genome where the *AGXT*_sg would bind. From these data, a list of 13 unique top sites for potential off‐target editing was compiled (Table 1). DNA samples from monoclonal iPSC lines were collected and used as a template for PCR to amplify the 13 loci in separate reactions (PCR primers in Table S1). The PCR products were purified, Sanger sequenced, and analyzed for potential off‐target editing using the web tool EditR [24].

**TABLE 1:** Off‐target details.

Name	Prediction software and order	Target sequence	PAM	Number of mismatches	Gene	Locus	Sanger from iPSC	ONT from fibroblasts
*On‐target*		*GCTTCAGGGAACTCTGCCAC*	*AGG*		*exon:AGXT*	*chr2: +240871427*	✓	✓
OT1	Benchling 1	GCATCAGGGATCTCTGCCAC	AGG	2	intergenic	chr18: −53844013	✓	✓
OT2	Benchling 2/IDT 1/CRISPOR 1	CTTTCAGGGTACTCTGCCAC	AGG	3	intron:AC007563.5	chr2: −216743330	✓	✓
OT3	Benchling 3	GGTTCAGAGAACTCTGCCAA	AAG	3	intergenic	chr11: +7524076	✓	✓
OT4	Benchling 4	ACCTCAGGGAACTCTGTCAC	AGG	3	intergenic	chrX: −53430342	✓	
OT5	Benchling 5	CCCGCAGGGCACTCTGCCAC	AGG	4	exon:C1orf109 (ENSG00000116922)	chr1: +37689798	✓	
OT6	IDT 2	GTCTCAGCTGAACTCTGCCAC	TAG	4	intergenic	chr11: −32306355	✓	✓
OT7	IDT 3	ACCTCAGTGAACTCTGCCAC	CAG	3	intergenic	chr9: −1576305	✓	✓
OT8	IDT 4	CCTCCAGGGAGCTCTGCCAC	AAG	3	intergenic	chr4: −6577799	✓	
OT9	IDT 5	ACTTGATGGAACTCTGCCTC	AGG	4	intergenic	chr5: −102226453	✓	
OT10	CRISPOR 2	TCTTCAGGGTGATCTGCCAC	GGG	4	intergenic:SH3GL2‐RP11‐570H19.2	chr9: +18132496–18132518	✓	✓
OT11	CRISPOR 3	GGTTTAGAGAACTTTGCCAC	AGG	4	exon:SFXN4	chr10: −119141159–119141181	✓	
OT12	CRISPOR 4	GGTTCTAGGAGCTCTGCCAC	AGG	4	intron:PLCG2	chr16: 81922193–81922215	✓	
OT13	CRISPOR 5	GGTGCAAGGAAATCTGCCAC	TGG	4	intron:PTPRD	chr9: −9395603–9395625	✓	

*Note:* This table shows the most likely off‐target editing loci for the *AGXT*_sg. Three web tools, Benchling, IDT, and CRISPOR, were used to predict the most likely off‐target loci across the genome where the *AGXT*_sg could bind. Selection of the top five sites from each software resulted in a list of 13 unique off‐target loci that were analyzed for potential off‐target editing from Sanger sequences of iPSC lines. The top six off‐target sites (OT1, −2, −3, −6, −7, and −10) were analyzed from ONT‐sequences of bulk edited fibroblasts. ✓, Off‐target analyzed.

Abbreviation: PAM, protospacer adjacent motif.

### 2.6 On‐ and Off‐Target Analysis From Oxford Nanopore Technology Sequencing

Genomic DNA was isolated from a bulk population of electroporated fibroblasts and used as a template to amplify the genomic locus around the *AGXT* variant or a predicted off‐target site in a PCR reaction (PCR primers in Table S1). The resulting PCR product was purified, and an equal amount of purified DNA from three replicate reactions was pooled for ONT sequencing. The sequence reads were analyzed for editing using the web tool CRISPResso2 [25]. The raw reads were aligned to reference amplicons using a minimum homology threshold of 60 for on‐target samples and 50 for off‐target samples. The aligned on‐target reads that had less than 1% representation of all aligned reads were excluded from analysis. All aligned off‐target reads were included in the analysis for increased sensitivity for rare off‐target events. The aligned off‐target reads were analyzed for the frequency of A>G‐ and C>T substitutions, and total transitions in the top six off‐target protospacers (Table 1).

### 2.7 Hepatocyte Differentiation

The iPSC lines were differentiated into hepatocyte‐like cells as previously described [22]. Briefly, the iPSCs were cultured in E8 medium (Thermo Fisher Scientific; A1517001) before differentiation. On the day before the start of the differentiation, cells were treated with 0.5 mM EDTA in PBS, resuspended into single cells with DMEM (Sigma; 6546), and seeded onto Matrigel‐coated (Corning, 356231) 12‐well plates (800000 cells per well) containing E8 and 10 μM ROCK inhibitor (Y‐27632; Selleckchem). On day 0, the medium was changed to a definitive endoderm induction medium consisting of Basal 1 media (MCDB 131 free from L‐Glutamine [PAN BIOTECH; P04‐80057] with 5 mg/mL Bovine Serum Albumin [Sigma; A7030], 1.5 mg/mL NaHCO3 [Sigma], Glucose and Glutamax) supplemented with 0.1 mg/mL Activin A (Qkine; Qk001), and 3 mM CHIR‐99021 (Tocris; 4423). On day 1, the medium was changed to Basal 1 media with 0.1 mg/mL Activin A and 0.3 mM CHIR. On day 2, the medium was changed to Basal 1 media with 0.1 mg/mL Activin A, and the definitive endoderm induction efficiency was estimated by flow cytometry using BD Pharmingen PE Mouse Anti‐Human CD184 (#555974) to stain a definitive endoderm marker CXCR4 and BD Pharmingen PE Mouse IgG1 (#555574) as a control. The differentiation was continued if the cell population reached a threshold of 80% CD184‐positive cells at this stage. From day 3 until day 20, a previously described hepatocyte differentiation protocol was followed [26].

### 2.8 Colocalization Analysis

To determine the level of AGT colocalization with mitochondria and peroxisomes, the differentiated, fixed, and immunostained cells were first imaged with a Leica SP8 X confocal microscope using 512 × 512 and 4096 × 4096 resolution formats for mitochondrial‐ and peroxisomal stainings, respectively. After confirming that each staining had worked with an overview of each slide and channel using low magnifications, Z‐stacks were captured at randomly selected locations along the microscope slides using the AGT‐stained channel for focusing before image capture. The “Overlay” view, where multiple channels can be viewed simultaneously, was not used before image capture. The Z‐stacks were deconvoluted using FIJI (ImageJ) software [27], and the deconvoluted images were analyzed for AGT‐TOMM20‐ and AGT‐PMP70 colocalization using the FIJI plugin Coloc2, which performs the pixel intensity correlation over space methods of Pearson and Manders. Costes' automatic thresholding was used for the Manders' method analyses. One to four regions of interest (ROI), each constituting roughly one cell positive for AGT accumulation, were manually selected for analysis from the AGT channel of each deconvoluted image. At least 12 independent ROI were analyzed for each of the three test conditions (Non‐edited, Edited, and Control).

### 2.9 Sample Preparation for Metabolomic Analysis

Cells were differentiated on Matrigel‐coated 12‐well plates with daily media changes until day 18, when the media was changed for the last time. The final media contained 0.5% ethylene glycol (Sigma; 324558) and a 50% decreased concentration of pyridoxine (4.3 μM pyridoxine hydrochloride) to challenge the cells. 48 h after the last media change, the media from all wells were collected and placed in separate tubes on ice. The cells were washed with cold PBS, detached, collected in 400 μL of +4°C extraction buffer (Acetonitrile/ddH2O 80:20) using a pipette, and transferred into a tube on ice. 50 μL of each media sample was transferred into a tube with 450 μL of +4°C extraction buffer. The diluted media and intracellular samples were vortexed for 10 s and centrifuged at 15800 RCF for 10 min at +4°C. 100 μL of the supernatant of each sample was transferred to a SureSTART 0.3 mL glass screw‐top micro vial (Thermo Scientific) and stored at −80°C for up to one month until the metabolomic analysis.

### 2.10 Metabolomic Analysis

The metabolomic analysis was performed as previously described [22]. Samples were analyzed on a Thermo QExactive Focus Quadrupole Orbitrap mass spectrometer coupled with a Thermo Dionex UltiMate 3000 HPLC system (Thermo Fisher Scientific). The high‐performance liquid chromatography system was equipped with a hydrophilic ZIC‐pHILIC column (150 × 2.1 mm, 5 μm, Merck Sequant) with a ZIC‐pHILIC guard column (20 × 2.1 mm, 5 μm, Merck Sequant). 5 μL of sample was injected into the liquid chromatography‐mass spectrometry instrument after quality controls in randomized order having every tenth sample as blank. A linear solvent gradient was applied in decreasing organic solvent (80%–35%, 16 min) at 0.15 mL min^−1^ flow rate and 45°C column oven temperature. Mobile phases were aqueous 200 mmol/L ammonium bicarbonate solution (pH 9.3, adjusted with 25% ammonium hydroxide), 100% acetonitrile, and 100% water. Ammonium bicarbonate solution was kept at 10% throughout the run, resulting in a steady 20 mmol/L concentration. Metabolites were analyzed using a mass spectrometer with a heated electrospray ionization source using polarity switching and the following settings: resolution of 70000 at m/z of 200; spray voltages of 3400 V for positive and 3000 V for negative mode; sheath gas of 28 arbitrary units (AU) and auxiliary gas of 8 AU; vaporizer temperature of 280°C; and ion transfer tube temperature of 300°C. The instrument was controlled using Xcalibur 4.1.31.9 software (Thermo Scientific). Metabolite peaks were confirmed using commercial standards (Sigma‐Aldrich). Data quality was monitored throughout the run using an in‐house quality control cell line extracted similarly to other samples. After final peak integration with TraceFinder 4.1 SP2 software (Thermo Scientific), peak area data were exported as Excel files. The absolute peak area of a metabolite of interest was normalized to the sum of the absolute peak areas of all 439 unique intracellular metabolites from the same sample well. Fresh media samples were normalized to the average sum of the absolute peak areas of all 439 intracellular metabolites from the healthy control wells.

### 2.11 LNP Formulation and Hepatocyte‐Like Cell Transfection

LNPs were formulated as previously described [22]. Briefly, the organic and aqueous phases were mixed in a volume ratio of 1:2 using the NanoAssemblr Spark (Precision Nanosystems), according to the vendor's instructions. The LNPs were immediately dispersed in a volume of neutral PBS buffer equal to the sum of the two input phases.

The organic phase consisted of SM‐102/DSPC/Cholesterol/DMG‐ PEG2000 (50/10/38.5/1.5 mol/mol). SM‐102 (BroadPharm CAT#: BP‐25499). Molar N/P: 6. The mixture was prepared as previously described [28, 29].

The aqueous phase consists of sodium citrate buffer (pH 4, 65 mM) containing the sgRNA and the ABE8e mRNA construct in a 1:2 mass ratio and with a total RNA concentration of 17 mg/mL.

The differentiating hepatocyte‐like cells on 12‐well plates were transfected with freshly prepared RNA‐LNP formulations by pipetting up to 2500 ng of total RNA into the differentiation media after a daily media change on day 19. The media was changed 24 h after, and the cells were collected for editing analysis five days after transfection.

### 2.12 Data Analysis

The output data from the different experiments was collected in tables and analyzed using GraphPad Prism version 9.3.1 for Windows, GraphPad Software, Boston, Massachusetts USA, www.graphpad.com. For multiple comparisons, we used one‐way ANOVA coupled with a Tukey test. Data are represented as the mean with SD.

## 3 Results

We aimed to model PH1 in vitro, specifically the effects of the Gly170Arg variant, and to correct the cell phenotype using base editors. We started by culturing fibroblasts from a skin biopsy of a compound heterozygous subject with variants c.508G>A (Gly170Arg) with minor allele c.32C>T (Pro11Leu), and c.673_676delAAGG. Next, we genetically corrected the Gly170Arg variant by electroporating the fibroblasts with in vitro transcribed ABEmax [30] mRNA and the Gly170Arg variant targeting single guide RNA. Sanger sequencing analysis of the editing outcomes with the EditR software [24] revealed that the ABEmax produced an average on‐target A‐T to G‐C editing efficiency of 98% without any unspecific editing along the protospacer sequence, validating the predicted potential of ABE to correct this variant (Figure 1B,C). To have a deeper insight into the editing outcomes, we sequenced the on‐target amplicons with Oxford Nanopore Technology (ONT) sequencing and analyzed the reads with CRISPResso2 software [25]. After editing, 86.7% of the reads that aligned to the reference amplicon showed a G base in position 6 encoding the healthy Gly170 variant without additional bystander edits in the protospacer, 6.1% of the reads showed an A base encoding the pathogenic Arg170 variant without bystanders, 5.6% a deletion, and 1.5% the healthy Gly170 with synonymous bystander edits that do not lead to additional amino acid changes (Figure 1D). To assess potential RNA‐guide‐directed off‐target base editing across the genome in the edited cells, we ONT‐sequenced amplicons from the top six off‐target loci (Table 1) predicted by three independent off‐target tools. The mean A>G, C>T, or total transition substitution rates across all sites were not higher for the edited samples in comparison to the non‐treated samples (Figure 1E). Additionally, we studied the on‐ and off‐target editing profile of a more processive ABE variant ABE8e [31] (Figure S1A–C), which produced equally high on‐target editing, but also significant editing in the nearby bystander bases.

Next, we generated corrected iPSC lines by simultaneously reprogramming and editing the patient fibroblasts with ABEmax, as previously described [21]. To assess the quality of the corrected cell lines and to screen out lines with potential off‐target edits, we sequenced 13 top off‐target loci (Table 1) predicted by three independent software programs. The Sanger sequencing data from these sites showed no A‐T to G‐C editing above a 1% threshold in the 54 adenines in the 13 potential protospacer regions where the guide could theoretically bind (Table S2). All patient‐derived iPSC lines expressed canonical pluripotency markers OCT4, NANOG, TRA1‐60, and SOX2 in immunostainings (Figure S1D).

**FIGURE 2:**
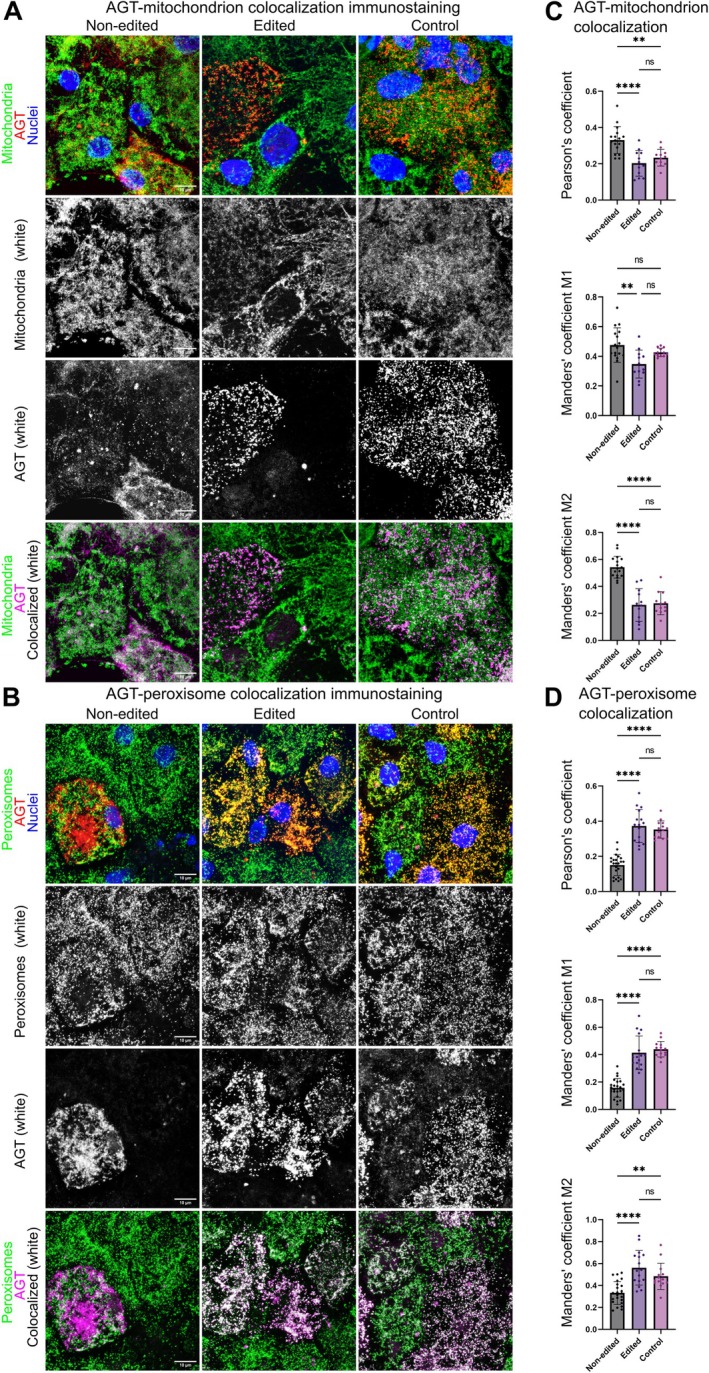
Gene editing of the Gly170Arg variant corrects enzyme mislocalization. (A and B) Representative immunocytochemistry images of day 20 hepatocyte‐like cells from Non‐edited‐(left), Edited‐(middle), and Healthy control (right) lines representing the level of AGT‐mitochondrion (A) and AGT‐peroxisome (B) colocalization. The top rows represent the nuclear stain Hoechst (blue), the mitochondrial label TOMM20 (green) (A) or the peroxisomal label PMP70 (green) (B), and AGT (red). The second and third rows show TOMM20 (A) or PMP70 (B) and AGT, respectively, in white. The bottom rows show TOMM20 (green) (A) or PMP70 (green) (B), AGT (magenta), and colocalized pixels with both TOMM20 (A) or PMP70 (B) and AGT in white. The white bar represents 10 μm. (C and D) Quantitative AGT‐mitochondrion (C) and AGT‐peroxisome (D) colocalization analysis performed with ImageJ (FIJI) software plugin Coloc2. The top graphs show Pearson's‐, the middle graphs Manders' M1‐, and the bottom graphs Manders' M2 coefficients for the colocalization of AGT and mitochondrion (C) or AGT and peroxisome (D). At least 12 regions of interest per test condition (Non‐edited, Edited, and Control), roughly constituting one cell positive for AGT accumulation, were manually selected for analysis from the AGT channel. Costes' automatic thresholding was used for the Manders' method analyses. Data are represented as the mean with SD. Statistical significance is based on the Tukey test: *p* > 0.05 (ns, not significant), *p* < 0.05 (*), *p* < 0.01 (**), *p* < 0.001 (***), *p* < 0.0001 (****).

To model the disease phenotype and the effects of ABE‐mediated Gly170Arg editing on the phenotype in vitro, we differentiated the iPSC lines into hepatocyte‐like cells using established protocols [26, 32] as shown previously [22]. At the end of the 20‐day differentiation protocol, the differentiated lines showed a robust expression of hepatic marker mRNA, such as *ALB*, *HNF1A*, *AFP*, and *APOA2* in comparison to the iPSC stage (Figures 1F and S2). The hepatic marker expression in the individual cell lines was comparable to the HepG2 hepatocellular carcinoma line, but did not reach the level of primary hepatocytes in *ALB* expression. Pluripotency marker *SOX2* expression decreased in the differentiated lines compared to the iPSC stage. *AGXT* expression increased significantly in all differentiated lines from the iPSC stage. The patient‐derived lines carrying one copy of the 673_676delAAGG allele showed approximately half of the *AGXT* expression levels of the healthy control line with two wild‐type *AGXT* alleles. All hepatocyte‐like cell lines showed an accumulation of hepatic markers AGT, HNF4A, and AFP in immunostainings (Figures 1G, 2A,B, and S2).

As the Gly170Arg variant has been shown to lead to AGT enzyme mislocalization, we determined the enzyme localization both in the non‐corrected and corrected hepatocyte‐like cell lines. After co‐staining the cells with anti‐mitochondrial and anti‐AGT labels, we observed AGT in non‐corrected cell lines colocalizing with mitochondria, whereas the corrected and control lines showed a different pattern and no co‐localization with the mitochondrial marker (Figures 2A and S2). Conversely, the immunostaining with anti‐peroxisomal and anti‐AGT labels showed co‐localization in the corrected and control lines, while the non‐corrected lines showed a different pattern (Figures 2B and S2). Quantitative analysis confirmed the pattern of mitochondrial enzyme localization in the non‐corrected lines and peroxisomal enzyme localization in the corrected and control lines (Figure 2C,D).

**FIGURE 3:**
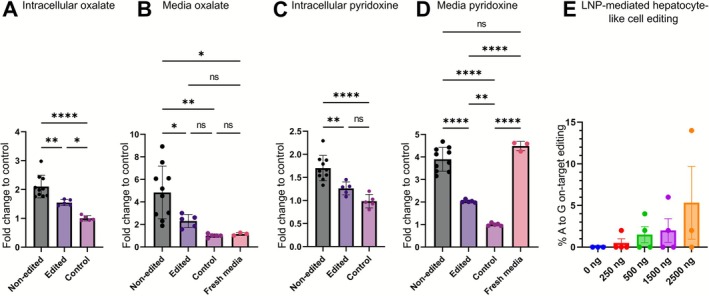
Metabolic profiling and in vivo applicable LNP‐mediated base editing. (A, B, C, and D) Metabolic analysis of differentiated hepatocyte‐like cell culture media detected by liquid chromatography‐mass spectrometry from different test conditions: Non‐edited (*n* = 2 independent cell lines), Edited (*n* = 1 cell line), and Control (*n* = 1 cell line). After a 48‐h incubation, cells and media from five replicate wells per cell line and three replicate samples of the fresh media were sampled for metabolic analysis. Relative abundance of (A) intracellular oxalate, (B) media oxalate, (C) intarcellular pyridoxine, and (D) media pyridoxine in relation to the control lines. Each sample was normalized to the sum of the peak areas of 439 metabolites detected in the same sample. Data are represented as the mean with SD. Statistical significance is based on the Tukey test: *p* > 0.05 (ns, not significant), *p* < 0.05 (*), *p* < 0.01 (**), *p* < 0.001 (***), *p* < 0.0001 (****). (E) The LNP mediated on‐target A to G editing from Sanger sequencing data of differentiated cells. Four doses of total RNA (250 ng, 500 ng, 1500 ng, and 2500 ng; synthesized single guide RNA and in vitro transcribed ABE8e mRNA in a 1:2 mass ratio) within LNP formulations were pipetted onto the culture media on day 19 of the differentiation. The media was changed 24 h after and the cells were collected for editing analysis five days after transfection.

To determine the metabolic state in the iPSC‐derived hepatocyte‐like cells, we used liquid chromatography‐mass spectrometry to evaluate the levels of the disease‐specific metabolites oxalate and pyridoxine in the cells and culture media after a 48‐h incubation (Figure 3A–D). The fresh cell culture media contained pyridoxine, which is a precursor of a co‐factor for AGT and significantly improves the function of the Gly170Arg variant [33]. To challenge the function of AGT, we reduced the concentration of pyridoxine by 50% and added glyoxylate precursor ethylene glycol [34] in the final media. Oxalate, the main diagnostic metabolite in PH1, was significantly elevated in the non‐corrected cell lines in comparison to the corrected and healthy control lines in both intracellular (Figure 3A) and media (Figure 3B) samples. The oxalate level in the corrected cell line remained moderately higher than in the control line both intracellularly and in the media. The non‐corrected lines had the highest, the corrected line the second highest, and the control line the lowest pyridoxine concentration in both the intracellular (Figure 3C) and the media (Figure 3D) samples after a 48‐h incubation.

After showing the efficient ABE‐mediated genetic correction of the *AGXT* Gly170Arg variant and the resulting restoration of the cell phenotype, we wanted to model potential hepatocyte editing in our in vitro model. We decided to employ a highly efficient ABE variant ABE8e [31] as mRNA and our Gly170Arg variant targeting single guide RNA encapsulated in LNP formulations using a strategy that we have optimized earlier to edit fibroblasts [22]. We added the formulations to the hepatocyte‐like cell culture media for 24 h on day 19 of the differentiation. In this reaction, we observed dose‐dependent on‐target editing reaching an average of 5% with the highest dose of 2500 ng of total RNA used (Figure 3E).

## 4 Discussion

In this study, we analyzed the effects of the most common PH1‐causing pathogenic *AGXT* variant using a relevant patient‐derived hepatocyte‐like cell model. We studied the effects of the precise gene correction on the cell phenotype, focusing specifically on enzyme localization and metabolism. In addition, we assessed the potential of base editors to correct this pathogenic variant with both in vitro‐ and in vivo–compatible delivery methods.

The achieved editing results corroborate the potential of ABE to correct the targeted variant efficiently and precisely. Utilizing electroporation and in vitro‐transcribed ABEmax mRNA, we reached a high on‐target mean editing efficiency of 98% without any proximal bystander editing in the protospacer region, confirmed by EditR analysis of the Sanger sequencing data. The ABE8e variant performed almost as efficiently as ABEmax on the target mutation, but produced more bystander edits. ONT sequencing analysis confirmed the successful on‐target editing, showing that the vast majority of the reads encoded for the healthy allele after the editing reaction. Analysis of the top *in silico* predicted off‐target loci showed no off‐target substitutions above the baseline of the non‐treated samples on average. In the edited iPSC clones, the *in silico* predicted off‐target sites 3 and 11 showed 1% editing in one position along the guide. We estimate that this editing is a background in the Sanger sequencing data. As the off‐target analyzed corrected iPSC lines were monoclonal in origin, a true off‐target editing event would show around 50% or 100% editing, respectively, if one or both alleles were edited. To model potential in vivo editing of the liver hepatocytes, we used the more processive ABE8e [31] mRNA encapsulated within LNPs to target the differentiating hepatocyte‐like cells. In this experiment, we reached a maximum of 5% mean on‐target editing without any bystander base changes. LNPs have been shown to preferentially accumulate in the liver and transfect hepatocytes through low‐density lipoprotein receptor (LDL‐R)‐mediated endocytosis [20]. However, our cell model does not fully replicate the authentic in vivo environment, including continuous perfusion of the liver and circulating apolipoprotein E, which facilitates LNP delivery to hepatocytes via LDL‐R [35], and fully mature primary hepatocytes presenting LDL‐R for LNP binding. Previous preclinical studies have shown high LNP‐mediated in vivo CRISPR editing of the liver in murine and non‐human primate models [36, 37, 38]. Additionally, a recent report already established amelioration of a severe metabolic phenotype in a carbamoyl‐phosphate synthetase 1 deficiency patient through LNP‐mediated base editing of the liver [39]. Hence, we assume that our editing system would yield substantially higher editing in vivo than we reached with the hepatocyte‐like cell model. This hypothesis could be tested in animal models. However, even modest editing efficiency establishes proof‐of‐concept that grants further investigation.

In this study, we showed that the correction of the Gly170Arg variant alone in the genetic background of AGT‐Mi leads to the rescue of AGT localization from mitochondria to peroxisomes. Since there is strong evidence that the root cause of pathogenesis caused by the variant is due to enzyme mislocalization [14], the genetically corrected cell line was expected to exhibit a rescue of the metabolic pattern. Indeed, the corrected cell line showed a significant decrease in oxalate compared to the non‐corrected counterparts, although not fully matching the level of the control. Several factors could explain this observation. First, the healthy control line has two fully functional wild‐type alleles, whereas the genetically corrected lines have one functional corrected allele and the c.673_676delAAGG variant, which results in a premature termination codon. qPCR of the hepatocyte‐like cells showed around 50% reduction of *AGXT* mRNA levels in the patient‐derived cell lines in comparison to the healthy control line (Figure 1E), which suggests that the deletion‐containing transcript might be subjected to nonsense‐mediated decay. This dose‐dependent reduction in enzyme activity could explain the difference in oxalate accumulation between the corrected and control lines. In addition, the cell model might not be robust enough to replicate the full metabolic pathway with all relevant enzymes fully present. The variation in differentiation and the resulting maturity of cells might lead to artificial differences in enzyme expressions and metabolite accumulation between cell lines and differentiation batches. Finally, our differentiation medium contains a significant pyridoxine supplement (Figure 3C), which could not be completely removed without an apparent decrease in cell viability. Pyridoxine, a precursor to the AGT co‐enzyme pyridoxal‐5′‐phosphate (PLP), has been shown to significantly improve the function of the 170Arg variant [33, 40], and the supplementation might lead to a decreased difference in the accumulation of oxalate between the 170Arg and 170Gly variants. Pyridoxine was supplemented in media in supraphysiological concentrations; normally, it is not measurable in human plasma [41, 42]. However, we cannot directly deduce the level of the active form, membrane‐impermeable PLP [43], based on the measured amount of pyridoxine in the differentiated cells and media. Taken together, rescuing AGT peroxisomal localization by gene editing ameliorates oxalate metabolism.

For PH1 management, hyperhydration and crystallization inhibitors are used as supportive treatments [5, 44]. Additionally, pyridoxine can be supplemented to responsive subjects who have mistargeting variants. Until recently, the only curative treatment has been liver transplantation or combined liver and kidney transplantation. Two recently developed novel therapeutics are based on RNA interference (RNAi) of mRNA encoding enzymes in the glyoxylate pathway. Lumasiran targets *HAO1* (MIM: 605023) encoding hydroxyacid oxidase (EC 1.1.3.15) and leads to a high accumulation of soluble glycolate and depletion of oxalate, and Nedosiran targets *LDHA* (MIM: 150000) encoding lactate dehydrogenase and prevents the final conversion of glyoxylate to oxalate in the hepatocyte cytosol (Figure 1A) [6]. Recent preclinical work targeting *HAO1* [45, 46, 47], and *LDHA* [48] with adeno‐associated virus (AAV) or LNP‐delivered CRISPR‐Cas9 in PH1 mouse and rat models presented a significant decrease in urinary oxalate accumulation. The CRISPR gene knockout strategy could deliver a permanent amelioration of the toxic oxalate accumulation with a single dose. However, potential off‐target effects of double‐stranded DNA breaks inducing CRISPR/Cas9 nucleases need to be carefully assessed for therapeutic applications, and the long‐term effects of elevated glycolate or glyoxylate need to be monitored. Strategies to treat PH1 through delivery of the human *AGXT* complementary DNA with viral vectors have been effective in restoring the metabolic phenotype in a mouse model [33] and AGT expression in a cell model of PH1 [49]. Recently, a human AGT mRNA‐Lipopolyplex therapy recovered functional enzyme expression and significantly reduced urinary oxalate in a rat model of PH1 [50]. As the in vitro differentiation protocols improve, autologous transplantations of genetically corrected iPSC‐derived hepatocyte‐like cells could provide a treatment for PH1 [49, 51]. The function and safety of these in vitro cultured cells need to be carefully assessed before reintroduction to patients; however, methods are being developed to make the cells safe for transplantation [52]. Our strategy to treat PH1 is based on CRISPR base editor mRNA and single guide RNA encapsulated within LNPs for liver targeting. This base editing approach corrects the mutant variant into a healthy one, a change that would be permanent and inherited by the daughter cells of proliferating liver hepatocytes. Furthermore, gene correction with base editing allows physiological regulation of expression, an advantage compared to the gene addition approaches. Unlike with the RNAi or mRNA replacement strategies, continuous redosing would not be necessary. However, compared to the viral vectors, LNPs are potentially less immunogenic, and redosing could be applied to target a higher proportion of edited hepatocytes. *HAO1*‐ and *LDHA‐targeting*, and *AGXT* gene or mRNA addition therapies could theoretically be applied to all PH1 subjects regardless of the *AGXT* variant they carry, whereas only 24%–37% of PH1 subjects carry the Gly170Arg variant [6]. Nevertheless, the base editing strategy could potentially be applied to most of the PH1 mutations. Data retrieved from the gnomAD database show that out of all *AGXT* variant alleles, which are annotated “Pathogenic”, “Likely pathogenic” or “Pathogenic/Likely pathogenic”, 74% are transition variant alleles that have the potential to be corrected with either ABE (69% of all alleles) or cytosine base editor (5% of all alleles) (Table 2). With just minor changes, it could be possible to target transition variant alleles other than Gly170Arg, switching only the single guide RNA, and when necessary, the type of base editor used. Additionally, the AGT‐Mi remains an alternative target for ABE. Several pathogenic variants associated with the mistargeting of AGT, including Gly170Arg, Ile244Thr, Phe152Ile, and Gly41Arg, are pathogenic only in the genetic background of the AGT‐Mi [53]. Thus, editing the AGT‐Mi into the major allele could rescue the phenotype of these four pathogenic variant alleles, collectively representing 58% of all pathogenic variant alleles, and the same genetic treatment could help most of the PH1 patients.

**TABLE 2:** Top 30 pathogenic *AGXT* variants.

ClinVar ID	Mutation	Protein consequence	Annotation	Clinical significance	Proportion of pathogenic *AGXT* alleles (%)	AGT‐Mi association	Mutation reversible with base editors
rs121908529	c.508G>A	p.Gly170Arg	Missense variant	Pathogenic/Likely pathogenic	47.0	Yes^a^	Yes (ABE)
rs180177201	c.33dup	p.Lys12GlnfsTer156	Frameshift variant	Pathogenic	7.6		No
rs121908524	c.454 T>A	p.Phe152Ile	Missense variant	Pathogenic	5.3	Yes^a^	No
rs121908523	c.121G>A	p.Gly41Arg	Missense variant	Pathogenic	4.4	Yes^a^	Yes (ABE)
rs180177286	c.847‐3C>G	c.847‐3C>G	Splice region variant	Pathogenic/Likely pathogenic	4.0		No
rs138025751	c.353G>A	p.Arg118His	Missense variant	Likely pathogenic	1.9		Yes (ABE)
rs180177156	c.1049G>A	p.Gly350Asp	Missense variant	Pathogenic/Likely pathogenic	1.6		Yes (ABE)
rs180177267	c.777‐1G>C	c.777‐1G>C	Splice acceptor variant	Pathogenic/Likely pathogenic	1.5		No
rs121908525	c.731 T>C	p.Ile244Thr	Missense variant	Pathogenic	1.4	Yes^a^	Yes (CBE)
rs180177239	c.568G>A	p.Gly190Arg	Missense variant	Pathogenic/Likely pathogenic	1.4		Yes (ABE)
rs121908526	c.697C>T	p.Arg233Cys	Missense variant	Pathogenic/Likely pathogenic	1.4		Yes (ABE)
rs180177191	c.28C>T	p.Pro10Ser	Missense variant	Pathogenic	1.2		Yes (ABE)
rs202108064	c.216C>G	p.Asn72Lys	Missense variant	Likely pathogenic	1.1		No
rs180177203	c.332G>A	p.Arg111Gln	Missense variant	Likely pathogenic	1.1		Yes (ABE)
rs121908527	c.698G>A	p.Arg233His	Missense variant	Pathogenic/Likely pathogenic	1.1		Yes (ABE)
rs180177166	c.116_117dup	p.Ala40GlnfsTer7	Frameshift variant	Pathogenic/Likely pathogenic	0.8		No
rs121908530	c.466G>A	p.Gly156Arg	Missense variant	Pathogenic	0.7		Yes (ABE)
rs180177195	c.302 T>C	p.Leu101Pro	Missense variant	Pathogenic/Likely pathogenic	0.7		Yes (CBE)
rs180177281	c.846 + 1G>T	c.846 + 1G>T	Splice donor variant	Pathogenic	0.6		No
rs180177173	c.139G>A	p.Gly47Arg	Missense variant	Pathogenic	0.6		Yes (ABE)
rs180177227	c.481G>A	p.Gly161Ser	Missense variant	Pathogenic	0.6		Yes (ABE)
rs180177230	c.497 T>C	p.Leu166Pro	Missense variant	Pathogenic	0.5		Yes (CBE)
rs180177264	c.757 T>C	p.Cys253Arg	Missense variant	Pathogenic/Likely pathogenic	0.5		Yes (CBE)
rs180177161	c.1079G>A	p.Arg360Gln	Missense variant	Pathogenic/Likely pathogenic	0.5		Yes (ABE)
rs180177162	c.107G>A	p.Arg36His	Missense variant	Pathogenic/Likely pathogenic	0.5		Yes (ABE)
rs180177207	c.346G>A	p.Gly116Arg	Missense variant	Pathogenic/Likely pathogenic	0.5		Yes (ABE)
rs767586362	c.175G>A	p.Glu59Lys	Missense variant	Pathogenic	0.4		Yes (ABE)
rs121908522	c.245G>A	p.Gly82Glu	Missense variant	Pathogenic/Likely pathogenic	0.4		Yes (ABE)
rs180177211	c.371A>C	p.His124Pro	Missense variant	Pathogenic	0.4		No
rs180177201	c.33del	p.Lys12ArgfsTer34	Frameshift variant	Pathogenic/Likely pathogenic	0.4		No

*Note:* This table shows the top 30 *AGXT* variants annotated “Pathogenic”, “Likely pathogenic”, or” Pathogenic/Likely pathogenic”, listed from the most common to the least common, retrieved from the gnomAD database (July 3rd 2024). The variant is noted “Yes” in the “Reversible with base editor” column if it is a transition point mutation (G>A, C>T, A>G, or T>C) that is targetable with base editing.

Abbreviations: ABE, adenine base editor; CBE, cytosine base editor.

^a^ The pathogenesis of the variant is associated with the minor allele background [53].

Although less immunogenic than viral vectors, base editors pose a risk of an immunogenic effect. In humans, there are preexisting humoral and adaptive immune responses to SpCas9 [54, 55], which is the main component of base editors, including the ABEmax and ABE8e variants used in this study. Off‐target DNA and RNA editing effects remain the other main risk factor with the base editors. The ABE poses an increased risk of RNA editing, since the deaminase component of ABE is evolved from tRNA adenosine deaminase [15]. In this preclinical study, we did not detect clear off‐target editing events in the top loci that are the most likely unspecific binding sites for the guide RNA. However, for clinical applications, the screening for potential off‐target editing sites would have to be extensive and genome‐wide.

Primary hyperoxaluria type 1 is a severe disease often leading to liver and kidney transplantation, a procedure with risks and requiring life‐long immunosuppressive medication. The development of gene therapy options can offer curative treatment for the disease in the future. Here, in this preclinical proof‐of‐concept study, we present a promising gene editing approach to correct *AGXT* mutations and successfully validate it in vitro.

## Author Contributions

Conceptualization: T.K, K.W., and M.E.H. Methodology: T.K., S.J., J.J., and E.K. Investigation: T.K., S.J, I.G., J.J., and N.K. Analysis and interpretation of data: T.K., S.J., I.G., J.J., N.K., E.K., V.H., D.B., K.W., and M.E.H; Visualization: T.K. Writing – original draft: T.K. and M.E.H. Writing – review and editing: T.K, S.J., I.G., J.J., N.K., E.K., V.H., D.B., K.W., and M.E.H. Resources: V.H., D.B., K.W., and M.E.H. Supervision: K.W. and M.E.H. Project administration: K.W., and M.E.H. Funding acquisition: K.W. and M.E.H.

## Funding

The work was funded by Helsinki University Hospital Research Funds, the Academy of Finland Centre of Excellence on Stem Cell Metabolism, the Foundation for Pediatric Research, the Paulo Foundation, the Magnus Ehrnrooth Foundation, the Research Council of Finland (grant 361593), the Novo Nordisk Foundation (NNF24OC0089232), and the Orion Research Foundation sr.

## Ethics Statement

The generation of fibroblast and hiPSC lines from skin biopsies was approved by the Coordinating Ethics Committee of the Helsinki and Uusimaa Hospital District upon informed consent of the donor or their guardians (diary no.: HUS/2754/2019). The study was conducted in accordance with the Declaration of Helsinki.

## Consent

Informed consent was obtained from the guardians of the subject involved in this study.

## Conflicts of Interest

The authors declare no conflicts of interest.

## Supporting information


**Data S1:** Supporting Information.

## Data Availability

The data that supports the findings of this study are available in the Supporting Information of this article.
