# Supplementary material for: Genetic Correction of the Most Common Mutation Causing Primary Hyperoxaluria Restores Enzyme Localization and Oxalate Metabolism
Source: J Inherit Metab Dis. 2025 Dec 2;49(1):e70122. doi: 10.1002/jimd.70122 (PMC12672195; doi:10.1002/jimd.70122)
Supplement: Supplementary file 1 — Data S1: Supporting Information. [file JIMD-49-0-s001.pdf]

Supplementary material

Supplementary Figure S1. ABE8e on- and off-target editing, and cell line validations.

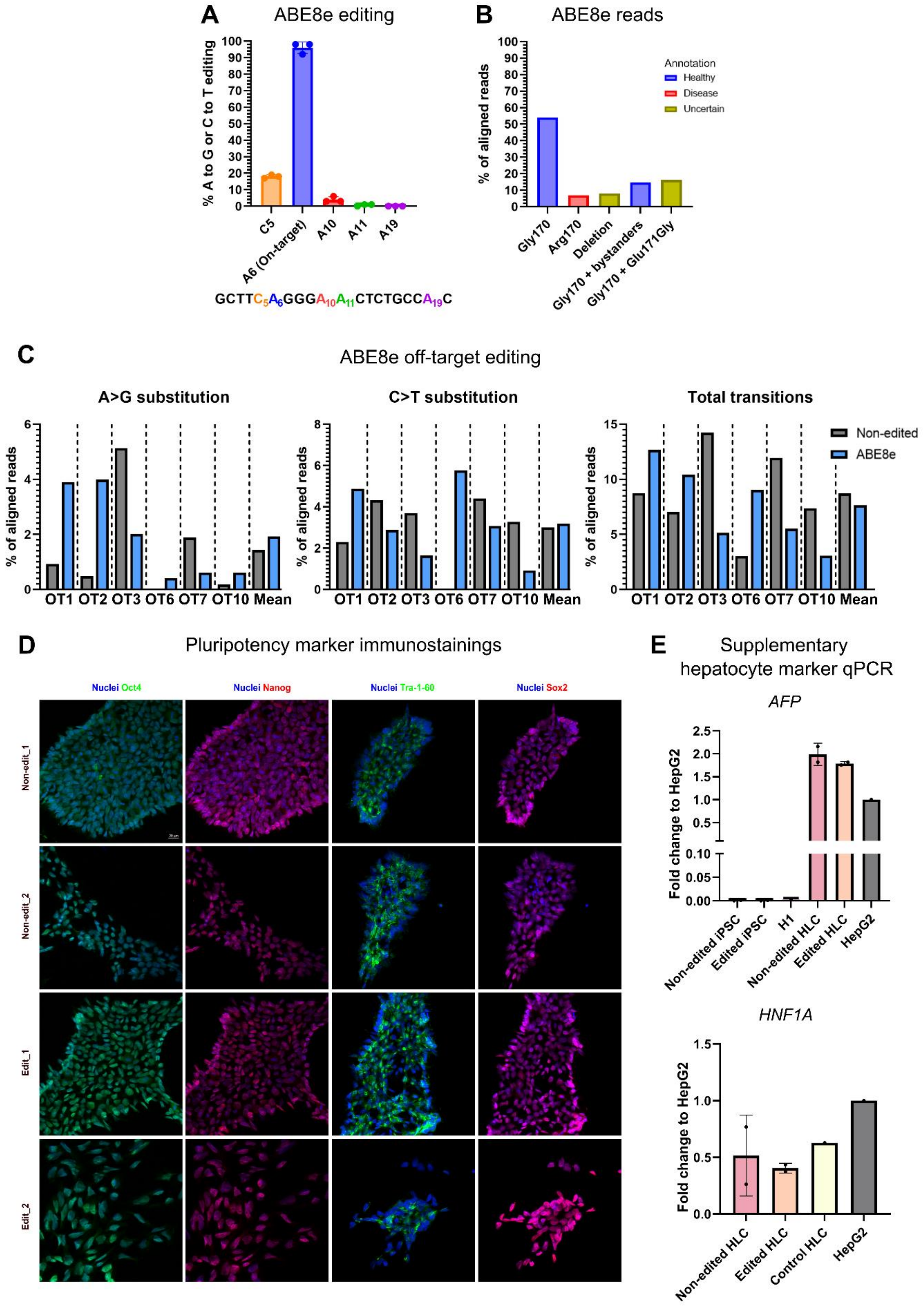

Figure S1. (A) ABE8e on-target editing. PCR amplicons from three replicate (n = 3) fibroblast electroporation reactions were Sanger sequenced and analyzed for ABE8e editing of AGXT c.508 and bystander bases using EditR. Data are represented as the mean with SD. (B) ABE8e ONT reads.The same electroporated DNA samples were pooled for ONT sequencing and analyzed with CRISPResso to get a deeper insight into the editing outcomes on an individual sequencing read level. The reads that aligned to the reference amplicon were analyzed based on the sequence in the 20 base pair long c.508 protospacer sequence and grouped into categories: “Gly170”, that includes all reads encoding the healthy Gly170 without additional base changes, “Arg170”, that includes all reads encoding the disease causing Arg170 without additional base changes, “Deletion”, that includes all reads with deletions, “Arg170 + bystanders”, that includes all reads encoding the healthy Gly170 with additional base changes that do not lead to additional aminoacid changes, and “Gly170 + Glu171Gly”, that includes reads that encode the Gly170 with additional base changes that lead to the Gly171Gly aminoacid change. The categories were annotated “Healthy”, “Disease”, and “Uncertain” based on the predicted amino acid sequence of the reads of the category. The mean read count aligned to the reference amplicon was 371 for the on-target samples. (C) Off-target editing of ABE8e. The electroporated DNA samples were pooled for ONT sequencing and analyzed for off-target editing with CRISPResso. All aligned off-target reads were included in the analysis for increased sensitivity for rare off-target events. The aligned reads were analyzed for the frequency of A>G substitutions, C>T substitutions, and total transitions (A>G, C>T, G>A, or T>C) in the top six off-target protospacers OT1, OT2, OT3, OT6, OT7, and OT10 (Table 1) predicted by three independent software. The mean read count aligned to the reference amplicon was 400 for the off-target samples. (D) iPSC immunostainings. Representative immunocytochemistry images of two non-corrected iPSC lines (Non-edit_1 and Non-edit_2) and two corrected iPSC lines (Edit_1 and Edit_2). Nuclear stain Hoechst is depicted in blue, pluripotency markers Oct4 and Tra-1-60 in green, and Nanog and Sox2 in red. All images of the same stain were acquired and processed with the same settings. The white bar represents 20 μm. (E) Supplementary hepatocyte marker qPCR. The graphs depict the mRNA levels of essential hepatocyte markers *AFP and HNF1A* in iPSC lines: Non-edited (n=2 independent cell lines), Edited (n=2 independent cell lines), commercial embryonic stem cell line H1 (n=1), differentiated hepatocyte-like cell (HLC) lines: Non-edited (n=2 independent cell lines), Edited (n=2 independent cell lines), and Control Hel24.3 cell line carrying two copies of the wild-type *AGXT* allele (n=1), HepG2 commercial hepatocarcinoma cell line (n=1), and primary hepatocyte line (n=1), analyzed by qPCR. The data are represented as a fold change to the HepG2 line. Data are represented as the mean with SD.

Supplementary Figure S2. Supplementary colocalization images.

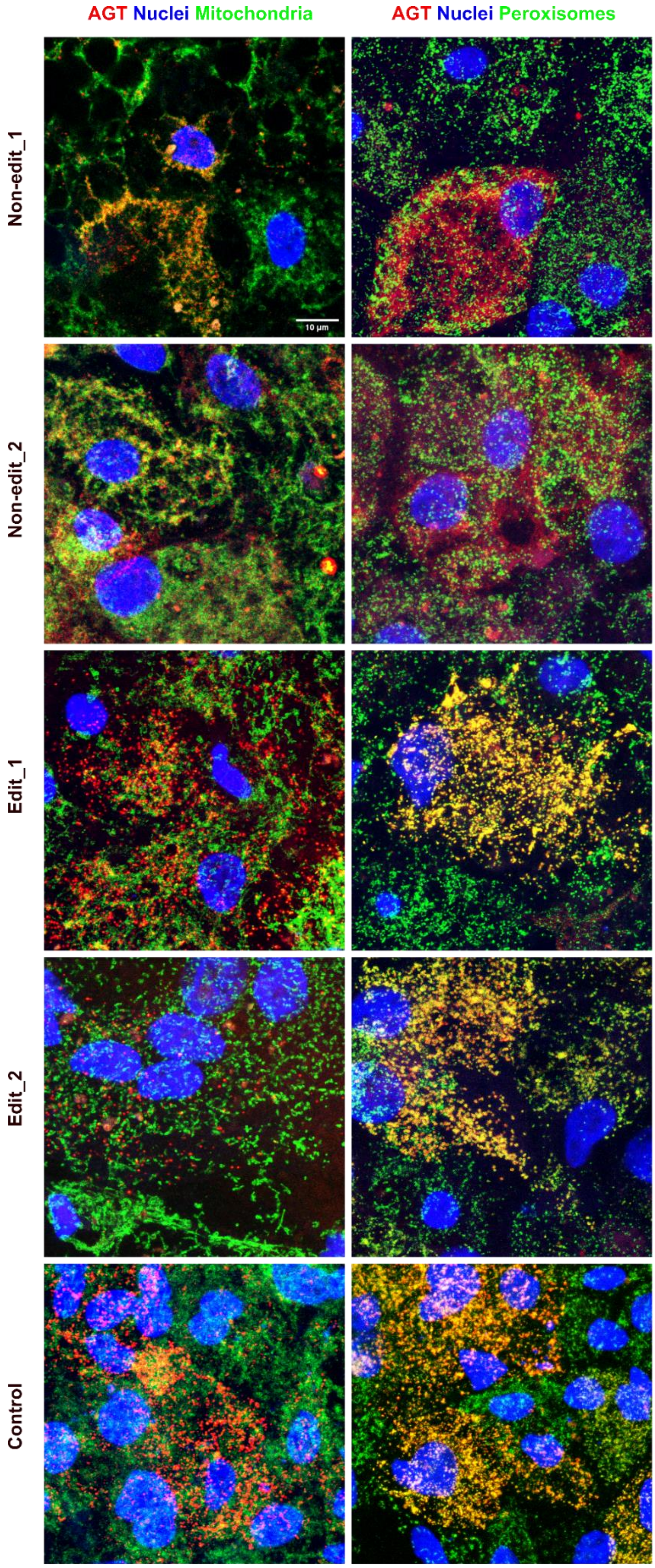

Figure S2 depicts representative immunocytochemistry images of day 20 hepatocyte-like cells from two non-corrected lines (Non-edit_1 and Non-edit_2), two corrected lines (Edit_1 and Edit_2), and healthy control line representing the level of AGT-mitochondrion (left panel) and AGT-peroxisome (right panel) colocalization. The nuclear stain Hoechst in blue, the mitochondrial label TOMM20 in green, and the peroxisomal label PMP70 in green. The white bar represents 10 µm.

Supplementary table S1. sgRNA and primers.

Name	Sequence	Function	Comment	Vendor
**Single guide RNA**				
sgRNA: AGXT_508	mG*mC*mU* rUrCrA rGrGrG rArArC rUrCrU rGrCrC rArCrG rUrUrU rUrArG rArGrC rUrArG rArArA rUrArG rCrArA rGrUrU rArArA rArUrA rArGrG rCrUrA rGrUrC rCrGrU rUrArU rCrArA rCrUrU rGrArA rArArA rGrUrG rGrCrA rCrCrG rArGrU rCrGrG rUrGrC mU*mU*mU* rU	Targets the *AGXT* c.508G>A variant in chromosome 2	RNA oligo	IDT
**PCR primers**				
AGXT_508_Fw	AGTCCATCTAGCAACAGCCC	Amplifies the genetic locus around the *AGXT* c.508G>A variant	DNA oligo	IDT
AGXT_508_Rv	GAGGTCAGTGGGCACTATGG	Amplifies the genetic locus around the *AGXT* c.508G>A variant	DNA oligo	IDT
AGXT_32_Fw	TCTCACCCCTGAGCTAAGCA	Amplifies the genetic locus around the *AGXT* c.32C>T variant	DNA oligo	IDT
AGXT_32_Rv	ATGCGGGTGGTATAGGGTGA	Amplifies the genetic locus around the *AGXT* c.32C>T variant	DNA oligo	IDT
OT1_Fw	TTTCTTGGGGGCTCCATTCC	Amplifies the genetic locus around a predicted off-target site	DNA oligo	IDT
OT1_Rv	CTTGGGACAGTGCTGCTTCT	Amplifies the genetic locus around a predicted off-target site	DNA oligo	IDT
OT2_Fw	CAGAGTAGCCCTTTGGTGGG	Amplifies the genetic locus around a predicted off-target site	DNA oligo	IDT
OT2_Rv	CCCCTCTCCCACAAACATCC	Amplifies the genetic locus around a predicted off-target site	DNA oligo	IDT
OT3_Fw	GGCTCATGAGGCCTCCTTTT	Amplifies the genetic locus around a predicted off-target site	DNA oligo	IDT
OT3_Rv	AATCGATCTCCACCCCCTCA	Amplifies the genetic locus around a predicted off-target site	DNA oligo	IDT
OT4_Fw	AGTACAGTACCCCGGCCATA	Amplifies the genetic locus around a predicted off-target site	DNA oligo	IDT
OT4_Rv	GCCATTCGTATGCAGCCTTG	Amplifies the genetic locus around a predicted off-target site	DNA oligo	IDT
OT5_Fw	CACTCCACACAAGCCGTACT	Amplifies the genetic locus around a predicted off-target site	DNA oligo	IDT
OT5_Rv	AAGTCATTGCGGCACTACGA	Amplifies the genetic locus around a predicted off-target site	DNA oligo	IDT
OT6_Fw	GCCTCCTCTGTCTTTCCCAC	Amplifies the genetic locus around a predicted off-target site	DNA oligo	IDT
OT6_Rv	ATTATGCTCCGGAAAGCCGT	Amplifies the genetic locus around a predicted off-target site	DNA oligo	IDT
OT7_Fw	GGCACTCCCTAGTGAGATGA	Amplifies the genetic locus around a predicted off-target site	DNA oligo	IDT
OT7_Rv	TCAGCCACCATTCTTTCCCC	Amplifies the genetic locus around a predicted off-target site	DNA oligo	IDT
OT8_Fw	CAGGAGAGCACGATCCCATC	Amplifies the genetic locus around a predicted off-target site	DNA oligo	IDT
OT8_Rv	CACTGGTTAAGGTCCCCGAC	Amplifies the genetic locus around a predicted off-target site	DNA oligo	IDT
OT9_Fw	TGGAAGAGGTGGGGCTGATA	Amplifies the genetic locus around a predicted off-target site	DNA oligo	IDT
OT9_Rv	TCCAGCAGTAGTGAGCACAT	Amplifies the genetic locus around a predicted off-target site	DNA oligo	IDT
OT10_Fw	TGTATGAGTGCTGCAGGGTC	Amplifies the genetic locus around a predicted off-target site	DNA oligo	IDT
OT10_Rv	CCCCTCACTGCCGATCTTAC	Amplifies the genetic locus around a predicted off-target site	DNA oligo	IDT
OT11_Fw	CAGGTTCGGATGGGTTGAGT	Amplifies the genetic locus around a predicted off-target site	DNA oligo	IDT
OT11_Rv	GGCATGGAGGTGCTTAGATG	Amplifies the genetic locus around a predicted off-target site	DNA oligo	IDT
OT12_Fw	TGGTTCACTGACCTCCTTGC	Amplifies the genetic locus around a predicted off-target site	DNA oligo	IDT

OT12_Rv	GAGGGCCTCATTGCTCTGTT	Amplifies the genetic locus around a predicted off-target site	DNA oligo	IDT
OT13_Fw	AATCCTGTGGGGAGTTGTGC	Amplifies the genetic locus around a predicted off-target site	DNA oligo	IDT
OT13_Rv	GCTCCCCAAGGGACTTCAAT	Amplifies the genetic locus around a predicted off-target site	DNA oligo	IDT
		**qPCR primers**		
AGXT_Fw	AGCCAGTGCTGCTGTTCTTA	quantifies AGT expression in qPCR	DNA oligo	IDT
AGXT_Rv	CCGGTCCATGTAAAGGGGG	quantifies AGT expression in qPCR	DNA oligo	IDT
AFP_Fw	CGCTGCAAACGATGAAGCAAG	quantifies AFP expression in qPCR	DNA oligo	Sigma
AFP_Rv	AATCTGCAATGACAGCCTCAAG	quantifies AFP expression in qPCR	DNA oligo	Sigma
ALB_Fw	GGAAAAGTGGGCAGCAAATGT	quantifies ALB expression in qPCR	DNA oligo	Sigma
ALB_Rv	GGTTCAGGACCACGGATAGA	quantifies ALB expression in qPCR	DNA oligo	Sigma
HNF1a_Fw	GGGCTTCTTGGACAACCTTTTCA	quantifies HNF1a expression in qPCR	DNA oligo	Sigma
HNF1a_Rv	CGTATGGACACCCGGCTCAT	quantifies HNF1a expression in qPCR	DNA oligo	Sigma
APOA2_Fw	ATGTGTGGAGAGCCTGGTTTCTCA	quantifies APOA2 expression in qPCR	DNA oligo	Sigma
APOA2_Rv	AAGCTCTGGGCTCTTGACCTTCT	quantifies APOA2 expression in qPCR	DNA oligo	Sigma
SOX2_Fw	GCCCTGCAGTACAACTCCAT	quantifies SOX2 expression in qPCR	DNA oligo	Sigma
SOX2_Rv	TGCCCTGCTGCGAGTAGGA	quantifies SOX2 expression in qPCR	DNA oligo	Sigma
CycloG_Fw	TCTTGTCAATGGCCAACAGAG	quantifies CycloG expression in qPCR	DNA oligo	Sigma
CycloG_Rv	GCCCATCTAAATGAGGAGTTG	quantifies CycloG expression in qPCR	DNA oligo	Sigma

Table S1 shows the sgRNA AGXT_508 employed to target the AGXT c.508G>A pathogenic variant. The table also shows the DNA primers for PCR and qPCR. The sequences are notated in 5'-to-3' orientation. In the "single guide RNA section", the protospacer is underlined, the non-underlined sequence is the canonical CRISPR-Cas9 tracrRNA. "r_" (rA, rC, rG, rU) refers to ribonucleic bases. "m_*" (mC*, mA*, mG*, mU*) refers to phosphorothioated 2'-O-methyl RNA bases.

Supplementary table S2. Off-target A>G editing.

Off-target	Off-target sequence	Non-edited	Edit_1	Edit_2
OT1	GCATCAGGGATCTCTGCCAC	0	0	0
OT2	CTTTCAGGGTACTCTGCCAC	0	0	0
OT3	GGTTCAGAGAACTCTGCCAA	0	1%, position 8	1%, position 8
OT4	ACCTCAGGGAACTCTGTCAC	0	0	0
OT5	CCCGCAGGGCACTCTGCCAC	0	0	0
OT6	GTCTCAGCTGAACTCTGCCAC	0	0	0
OT7	ACCTCAGTGAACTCTGCCAC	0	0	0
OT8	CCTCCAGGGAGCTCTGCCAC	0	0	0
OT9	ACTTGATGGAACTCTGCCTC	0	0	0
OT10	TCTTCAGGGTGATCTGCCAC	0	0	0
OT11	GGTTTAGAGAACTTTGCCAC	0	1%, position 8	0
OT12	GGTTCTAGGAGCTCTGCCAC	0	0	0
OT13	GGTGCAAGGAAATCTGCCAC	0	0	0

Table S2 shows the off-target A to G editing in the 13 *in silico* predicted off-target sequences in two independent corrected patient-derived iPSC lines represented as Edit_1 and Edit_2. One non-corrected patient-derived iPSC line ("Non-edited") serves as a control that has not been treated with base editor. The sequence column shows the protospacer sequence of the off-target. All adenine bases that are potential off-targets are highlighted in red. The off-target PCR amplicons were Sanger sequenced and analyzed with the EditR tool for potential editing. The highlighted boxes show the percentage of editing and the position along the off-target sequence where the A to G editing took place. 0 = no A to G editing in any of the adenine bases along the off-target protospacer sequence.

Supplementary table S3. Antibodies used.

Primary antibody	Raised in	Dilution	Vendor	Catalog number	Comment
OCT4	goat	1 to 500	Santa Cruz	sc-8628	Pluripotency marker
TRA-1-60	mouse	1 to 500	Thermo	MA1-023	Pluripotency marker
Nanog	rabbit	1 to 500	Cell Signalling Technology	D73G4	Pluripotency marker
SOX2	rabbit	1 to 500	Cell Signalling Technology	3579	Pluripotency marker
AGXT	rabbit	1 to 200	Sigma	HPA035371	Enzyme of interest/ hepatocyte marker
TOMM20	mouse	1 to 500	Abcam	ab56783	Mitochondrial marker
PMP70	mouse	1 to 500	Sigma	SAB4200181	Peroxisomal marker
AFP	rabbit	1 to 300	Agilent	A000829-2	Hepatocyte marker
Albumin	mouse	1 to 33	R&D Systems	MAB1455	Hepatocyte marker
HNF4A	rabbit	1 to 500	Cell Signalling Technology	3113	Hepatocyte marker
**Secondary antibody**	**Reactivity**	**Dilution**	**Vendor**	**Catalog number**	**Comment**
Alexa Fluor 488	anti-goat	1 to 500	Invitrogen	A11055	Green fluorescent
Alexa Fluor 488	anti-mouse	1 to 500	Invitrogen	A21202	Green fluorescent
Alexa Fluor 488	anti-rabbit	1 to 500	Invitrogen	A21206	Green fluorescent
Alexa Fluor 594	anti-mouse	1 to 500	Invitrogen	A21203	Red fluorescent
Alexa Fluor 594	anti-rabbit	1 to 500	Invitrogen	A21207	Red fluorescent

Table S3 compiles a list of primary and secondary antibodies used for immunocytochemistry in this study.

Supplementary methods

Cell culture

Fibroblasts: The skin biopsy sample was collected from a subject carrying *AGXT* variants c.508G>A (Gly170Arg), c.32C>T p.Pro11Leu, and c.673_676delAAGG in heterozygosity, manually sliced into pieces, seeded, and cultured under glass coverslips in a 60 mm dish. The culture medium consisted of DMEM (Sigma; 6546) supplemented with 20 % fetal bovine serum (FBS, Life Technologies; 10106-169), 1X GlutaMAX (Life Technologies; 35050-038), and 1 % penicillin-streptomycin (Life Technologies; 15140-122) until the fibroblast cells formed a monolayer, after which the cells were passaged with TrypLE^{TM} Select Enzyme (Thermo Fisher Scientific; 12563029). After passaging the fibroblasts from the plates containing the skin biopsies, they were cultured in DMEM supplemented with 10 % FBS and 1X GlutaMAX.

iPSC: The iPSC lines were cultured on Matrigel-coated (Corning; 356231) 6-well plates with Essential 8 medium (E8, Thermo Fisher Scientific; A1517001) and passaged with 0.5 mM EDTA in PBS after reaching 70 % confluency. The lines were employed for experimentation and differentiation after passage 15. HEL24.3 iPSC line[1] with two copies of the wild-type *AGXT* was used as a healthy control in the experiments.

HepG2 and primary human hepatocytes: HepG2 (Knowles BB, Aden DP. US Patent 4,393,133 dated Jul 12, 1983) is a commercial cell line isolated from a hepatocellular carcinoma of a 15-year-old, white male[2]. The cells were cultured in 12-well plates with no coating. Cryopreserved primary human hepatocytes (HEP187; Biopredic International) were thawed and cultured for 24 hours on Matrigel-coated 12-well plates before RNA collection for qPCR. The culture medium for HepG2 and

primary human hepatocytes consisted of DMEM supplemented with 10 % FBS, 1X GlutaMAX, and 1 % penicillin-streptomycin.

Immunocytochemistry

iPSCs were cultured until passage 15-20 in E8 medium (Thermo Fisher Scientific; A1517001) and seeded on glass coverslips on 24-well plate wells with Matrigel-coating (Corning; 356231). When 50 % confluency was reached, the cells were fixed.

iPSCs were differentiated for 20 days on glass coverslips on 24-well plate wells with Matrigel-coating before fixing.

The cells were fixed with 4 % PFA in PBS for 15 minutes, permeabilized with 0.5 % Triton X-100 in PBS for 15 minutes, and incubated with a blocking solution of 5 % BSA and 0.1 % Tween20 in PBS for one hour on a Stuart SSL4 seesaw rocker. They were incubated with primary antibody solution for 24 hours at 4°C on the seesaw rocker. The cells were treated with Hoechst 33342 (Thermo Fisher Scientific) and secondary antibody solution in the dark at room temperature for 30 minutes on the seesaw rocker. Before mounting on microscope slides, the cells were washed three times for 15 minutes in PBS on the seesaw rocker. Hoechst 33342 and all primary and secondary antibodies were diluted in a blocking solution containing 5 % BSA and 0.1 % Tween20 in PBS. The primary and secondary antibodies used are compiled in Table S3.

Supplementary references

1. Trokovic R, Weltner J, Otonkoski T. Generation of iPSC line HEL24.3 from human neonatal foreskin fibroblasts. *Stem Cell Res*. 2015;15(1):266-268. doi:10.1016/j.scr.2015.05.012

2. Aden DP, Fogel A, Plotkin S, Damjanov I, Knowles BB. Controlled synthesis of HBsAg in a differentiated human liver carcinoma-derived cell line. *Nature*. 1979;282(5739):615-616. doi:10.1038/282615a0
